# Determination of permeation properties of hydrogen gas in sealing rubbers using thermal desorption analysis gas chromatography

**DOI:** 10.1038/s41598-021-96266-y

**Published:** 2021-08-24

**Authors:** Jae Kap Jung, In Gyoo Kim, Ki Soo Chung, Yong-Il Kim, Dae Ho Kim

**Affiliations:** 1 Hydrogen Energy Materials Research Center, Korea Research Institute of Standards and Science, Daejeon, 34113 Korea; 2 ICT Creative Research Laboratory, Electronics and Telecommunications Research Institute, Daejeon, 34129 Korea; 3 Department of Physics, Research Institute of Natural Science, Gyeongsang National University, Jinju, 52828 Korea; 4 Division of Physical Metrology, Korea Research Institute of Standards and Science, Daejeon, 34113 Korea

**Keywords:** Energy science and technology, Materials science, Mathematics and computing, Physics

## Abstract

Permeation properties of hydrogen gas (H_2_) into nitrile butadiene rubber (NBR), ethylene propylene diene monomer (EPDM), and fluoroelastomer (FKM) which are the strong candidates for sealing material in H_2_ energy infrastructures, was quantified using a thermal desorption analysis gas chromatography (TDA GC) and a self-developed diffusion-analysis program. The samples were charged with H_2_ in a high-pressure chamber for 24 h then decompressed into atmosphere, and the mass of H_2_ released from the sample was measured as a function of elapsed time after decompression. The developed program calculated the total charging amount *C*_0_ and diffusivity *D*, which were then used to calculate the H_2_ solubility *S* and permeability *P* for variation of pressure. The samples were polymerized with and without carbon black (CB) filler in cylindrical shapes with different diameters. There was no appreciable pressure up to 12 MPa or diameter dependence investigated in this study on D, S and P. NBR and EPDM showed dual hydrogen diffusion with fast and slow diffusion behaviors caused by CB, whereas FKM showed a single diffusion behavior. The determined *D* are *D*_fast, NBR_ = (1.55 ± 0.28) × 10^–10^ m^2^/s, *D*_slow, NBR_ = (3.1 ± 0.5) × 10^–11^ m^2^/s, *D*_fast, EPDM_ = (3.65 ± 0.66) × 10^–10^ m^2^/s, *D*_slow, EPDM_ = (3.3 ± 0.5) × 10^–11^ m^2^/s, *D*_FKM_ = (7.7 ± 0.8) × 10^–11^ m^2^/s. It appeared that the filler contributes to increase *S* and decrease *D*. The uncertainty analysis against the evaluated data was carried out, too, in order that the method could be applicable as a standard test for the permeation properties of various polymer membranes.

## Introduction

Elastomer are utilized as gaskets for valves and pipelines in the H_2_ environment such as hydrogen stations. Examples include nitrile butadiene rubber (NBR), ethylene propylene diene monomer (EPDM), and fluoroelastomer (FKM) rubbers^1–5^. In this environment, the polymers are contacted with H_2_ under high pressure (HP) and their ability to withstand it is quantified. Previous research^6–10^ has considered physical parameters such as thermal property, volume expansion and glass transition, mechanical characteristics such as tension, compression, elastic modulus and hardness, and permeation parameters of H_2_.

The H_2_ can easily adsorb organic substances like as plastics and rubbers and lead change in physical stabilities^11^. Rubbery O ring is utilized to seal the H_2_ gas under HP in hydrogen fueling stations. The rubber seals are used in compressors, dispensing hoses, flange connections, and various valves in HP H_2_ infrastructure that regulate the charge/discharge of H_2_ from gas storage tanks^12–14^. Rubbers are critical fittings to ensure safety by preventing H_2_ leak and overcoming poor surroundings contact with high pressure H_2_ in the temperature region of − 40 °C to 90 °C. To discover suitable materials with low H_2_ permeability and with high endurance against such harsh H_2_ surroundings, determinations of H_2_ transport parameters are important, and a delicate investigation technique of the transport properties that clarifies H_2_ adsorption and diffusion in rubber is needed.

There are diverse methods to determine the permeability properties of gas such as gravimetric techniques^15^, magnetic suspension balance method^16,17^, manometric methods^18,19^, constant pressure methods^20^, differential pressure method^21^, carrier gas methods^22^ and computer modelling^23,24^. In this study, we established a quantitative ex situ thermal desorption analysis-gas chromatography (TDA-GC) method with the help of developed a diffusion-analysis program to evaluate permeation parameters such as H_2_ solubility *S*, diffusivity *D*, and permeability *P*. This paper is focus on set up and establishment of the gas permeation evaluation procedures by an application of diffusion law through TDA-GC. The TDA-GC method is appropriate for the elaborate analysis of H_2_ diffusion with dual components owing to better resolution of less than 0.1 wt·ppm. Thus, the role of filler contained the rubber could be assigned from the deconvolution of hydrogen content versus elapsed time after decompression. We evaluated the uncertainty of this method according to the Guide to the Expression of Uncertainty in Measurement (GUM) in order to set up a test protocol for hydrogen gas permeation within rubbery polymer materials^25^. For validation of the proposed method, the permeation parameters obtained in this study were compared with those by the other groups^26,27^.

## Gas diffusion model in cylindrical samples

H_2_ dissolved in rubber under HP is released into the air due to the pressure difference when the pressure vessel is opened. Assuming that the H_2_ is initially distributed uniformly in a cylindrical rubber sample and diffuses into an air, the change in the hydrogen gas residue *C*_*R*_ in the samples is a function of time *t* and expressed as follows^28^

1 $${C}_{R}(t)=\frac{32}{{\pi }^{2}}\times {C}_{0}\times \left[\sum_{n=0}^{\infty }\frac{exp\left\{{-(2n+1)}^{2}{\pi }^{2}Dt/{l}^{2}\right\}}{{(2n+1)}^{2}}\right]\times \left[\sum_{n=1}^{\infty }\frac{exp\left\{-D{\beta }_{n}^{2}t/{\rho }^{2}\right\}}{{\beta }_{n}^{2}}\right],$$

where *C*_0_ is the total charged amount of H_2_ into rubber in unit of [wt·ppm], *n* the number of the terms, $l$ the thickness of the cylindrical rubber sample, $\rho$ the radius of the sample, and ${\beta }_{n}$ the root of the zeroth-order Bessel function. Equation (1) is the solution of Fick’s second law of diffusion for a cylindrical sample^29^. The *D* and ${C}_{O}$ are obtained by Eq. (1) from the experimental ${C}_{R}(t)$ data.

The derivative of Eq. (1) at $t=0$, $\frac{d{C}_{R}(t=0)}{dt} = -\infty$, which means that the initial escape rate of H_2_ is very high. This phenomenon is due to an extreme distribution of H_2_ caused by the discontinuous pressure difference between the HP inside the rubber and the atmosphere pressure on the outside at $t=0$. Only the first two or three terms of summations in Eq. (1) are needed when $t$ is large enough (i.e. > 1 s). However, at small $t$, for example, below 0.1 s, we need more terms for converged values of the summation in the equation . At least five or more terms (*n*) are required, and a dedicated program is necessary for the precise analysis. We thus developed a diffusion analysis program that can calculate the *D* and ${C}_{O}$ from the experimental data, including up to the 50th summation terms of both brackets of Eq. (1).

**Figure 1:**
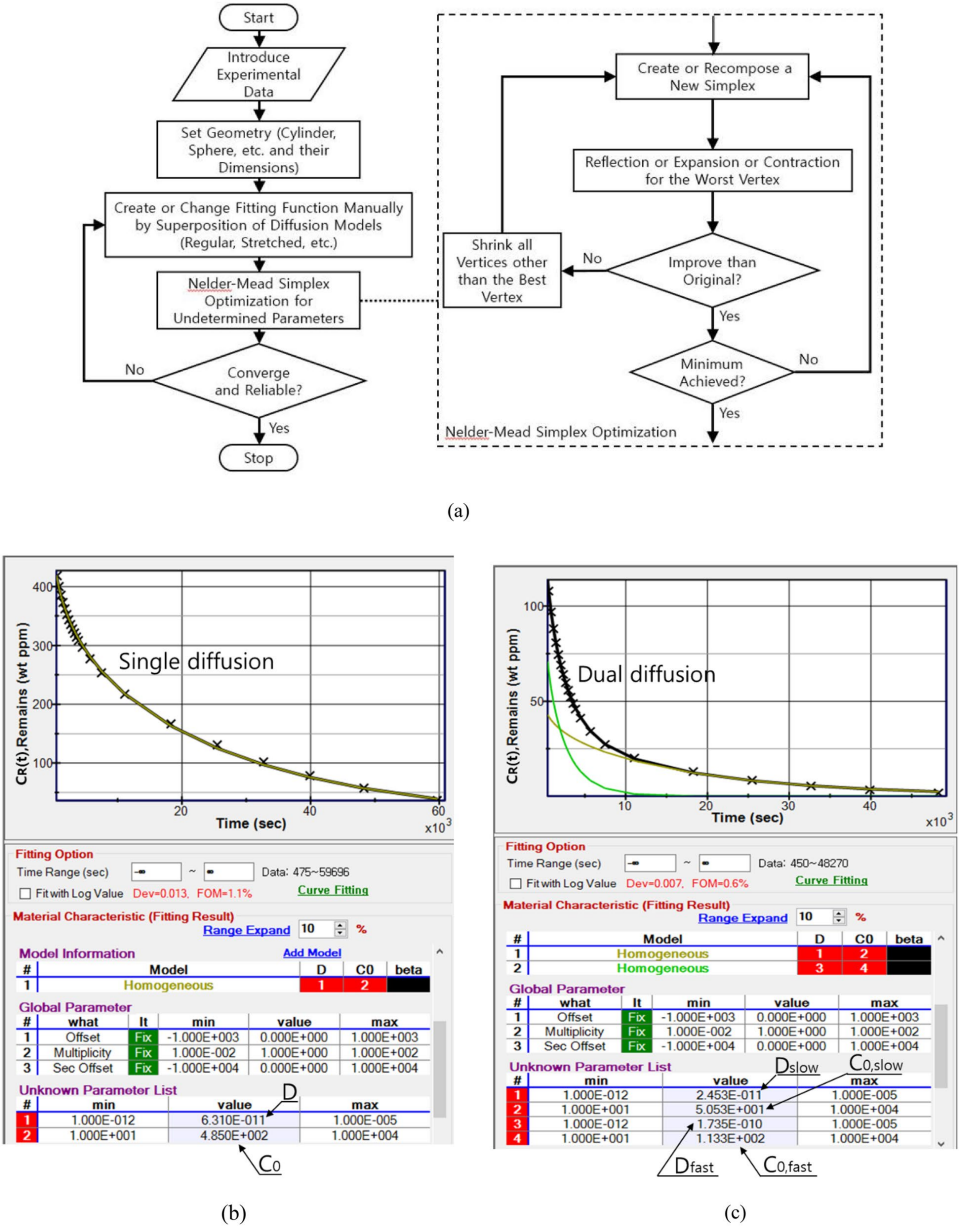
(**a**) Flow chart explaining the algorithm of the diffusion analysis program, application results of the program to obtain the ${C}_{O}$ and *D* by least squares regression for (**b**) single diffusion and (**c**) dual diffusion.

We show the flow chart explaining the algorithm of the developed program (Fig. 1a) by employing Nelder-Mead simplex optimization and an application examples of this program (Fig. 1b, c). The program was designed to be able to handle spherical and cylindrical shaped samples with different volume sizes. The black line and x of Fig. 1b indicate the line fitted with single Eq. (1) and experimental data, respectively. The data of H_2_ residue was fitted by least squares regression using optimizing the algorithm (Fig. 1b). In this example, the process yielded *D* = 6.31 × 10^–11^ m^2^/s and *C*_*0*_ = 485 wt·ppm (Fig. 1b). The C_0_ corresponds to the value at t = 0 by extrapolating the fitted line. The standard deviation between experimental date and fitted line was 1.1%. The fitted results yield $S=\frac{C}{p}$^30^ and $P=D\cdot S$^31^. Whereas, the result of Fig. 1c shows dual diffusion behaviors fitted with two Eqs. (1) by the application of the diffusion program. The black line is the sum of two yellow lines and x is the experimental data. Thus, we determine the values of D_fast_, C_0,fast_ and D_slow_, C_0,slow_ by diffusion program.

## Experiments and analyses

### Sample preparation

NBR is a synthetic rubber which is copolymerized with combinations of butadiene (CH_2_CH=CHCH_2_) and acrylonitrile (CH_2_CHCN). It is widely used as a sealing material due to its excellent gas resistance^32^, especially as an O-ring seals for flange connections, threaded connectors, and various valves in HP H_2_ infrastructure. The NBR samples used in this work are commercial ones synthesized with 50 wt % carbon black (CB) as a filler.

EPDMs are a type of synthetic rubber. EPDM elastomers have excellent resistance to heat, ozone, weathering, and aging^33^. These elastomers also exhibit excellent electrical insulation and low-temperature properties but only fair physical strength properties. EPDMs can be used in a wide range of applications, which typically include radiators, heater hoses, windows, door seals, O-rings and gaskets, accumulator bladders, wire and cable connectors, insulators, diaphragms, and weather stripping. Carbon black of 34%was included as a filler during fabrication of the EPDM specimen.

FKM is a fluorocarbon-based synthetic rubber made by copolymerizing tetrafluoroethylene (TFE), vinylidene fluoride (VF_2_) and hexafluoropropylene (HFP). This fluorinated elastomer has outstanding resistance to oxygen, ozone, and heat and to swelling by oils, chlorinated solvents, and fuels^34^. Carbon black of 14% was included as a filler during fabrication of the FKM specimen. The chemical compositions and properties or function of the three rubbers are shown in Table 1.

**Table 1:** Chemical compositions (according to function) and related properties of NBR, EPDM and FKM specimens.

Function or property	NBR	EPDM	FKM
Polymer	NBR (40)*	EPDM (58)	FKM (82)
Filler-reinforcing	Carbon black (50)	Carbon black (34)	Carbon black (14)
Processing aid	1,2-Benzenedicarboxylic acid (6)		
Antioxidant	2-Benzimidazolethiol (2)		
Curing agent	Sulfur (2)	Zinc oxide (3) Dicumyl peroxide (5)	Calcium dihydroxide (4)
Crystallinity^+^ (%)	0.24	3.97	0
Density (g/cm^3^)	1.30	1.15	1.89

*Numbers in ( ) are weight ratios in %.

^+^Degree of crystallinity (%) can be determined from area of melting peak by differential scanning calorimeter (DSC)^35^.

### TDA-GC measurement

**Figure 2:**
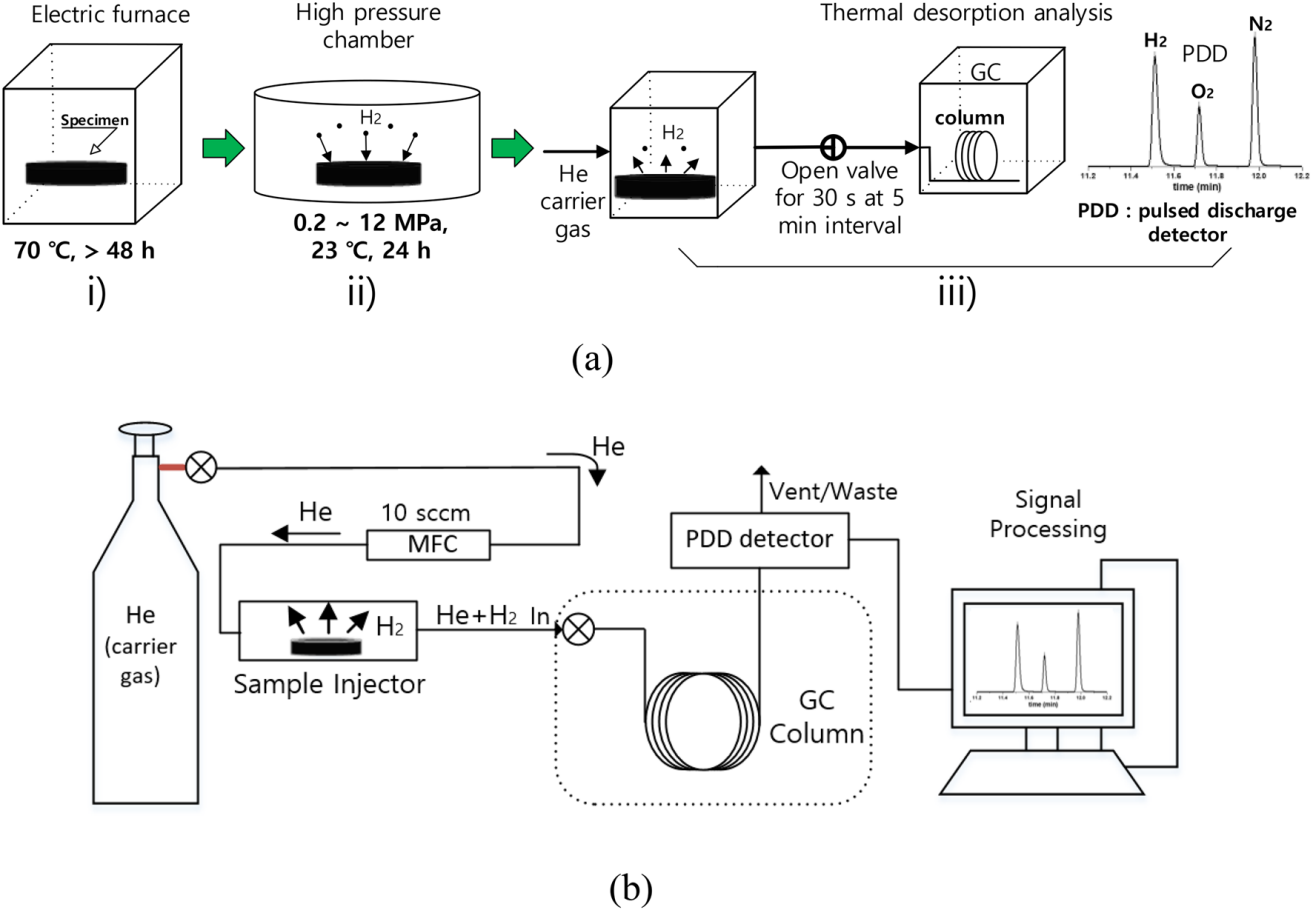
(**a**) Schematic diagram of entire TDA-GC measurement procedure, (**b**) configuration of TDA-GC measurement.

The TDA-GC (Agilent 7890 A) procedure (Fig. 2a) includes preliminary processes before measuring the rubber sample. First, the rubber was heat-treated (Fig. 2a, i) at 70 ℃ for at least 48 h, as recommended in CHMC 2^36^ to minimize outgassing from the rubber. Then it was charged with H_2_ at room temperature and the desired pressure for 24 h (Fig. 2a, ii); this duration was found to be sufficient to attain equilibrium in H_2_ sorption. The pressure was then lowered to atmospheric pressure by opening the exhaust valve of high pressure chamber, and the specimen was removed and loaded into a quartz tube (inner diameter of 14 mm and length of 60 mm) connected to GC injector to start measurements (Fig. 2a, iii). The elapsed time is recorded from the moment (*t* = 0) at which the HP hydrogen gas vessel is reduced to atmospheric pressure. The time lag between decompression and the start of TDA-GC measurement amounted to approximately 9 min.

**Figure 3:**
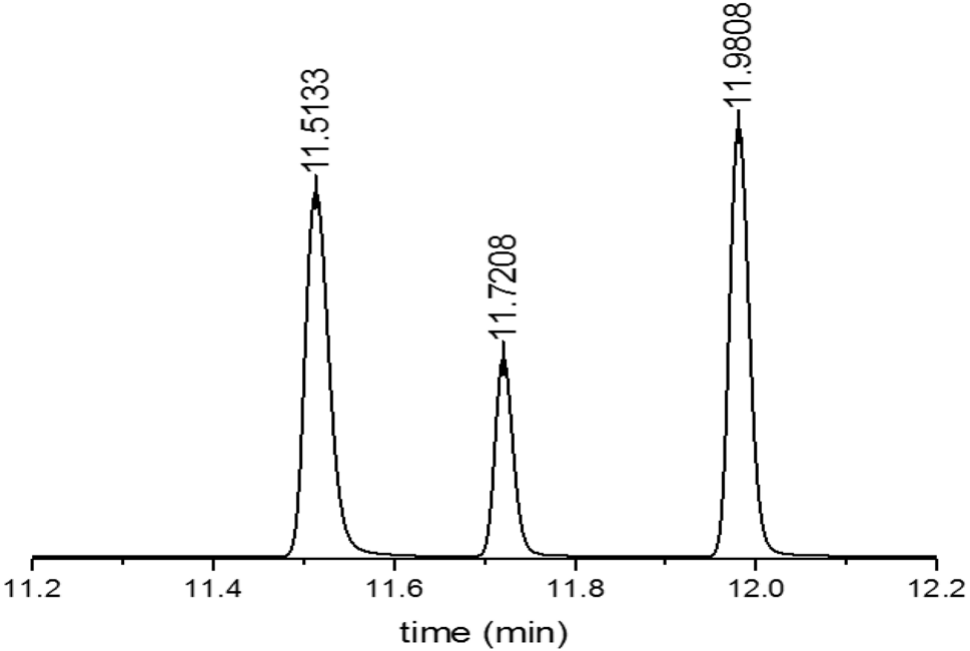
Typical GC spectra (peaks left to right: H_2_, O_2_, N_2_; units of peak height: pA) measured by an injection at 10 min after decompressing an NBR sample (cylindrical, diameter: 11.5 mm, thickness: 5.3 mm) charged with HP H_2_ for 24 h at 9 MPa.

TDA-GC analyzes the corresponding gas qualitatively and quantitatively by measuring the position and area of the separated GC signals^37^. The configuration (Fig. 2b) of a TDA-GC measurement is a detailed process of Fig. 2a, iii. The flow rate of helium (carrier gas) is controlled using a mass-flow controller (MFC)^38^. The gas released from the sample is mixed with the carrier gas and sent to the capillary GC column (inner diameter of 0.32 mm and length of 30 m) through the injector. Then, a pulsed discharge detector (PDD) produces electrical signals corresponding the separated gas components. At 0.1, 3, 5, 10, …, 990 min after decompression, the injection valve opened for 30 s and the hydrogen gas signal appeared at about 1.5 min after each injection (Fig. 3). Oxygen and nitrogen signals are not emitted by the rubber but are temporarily observed initially because of contact with air (containing the two gases) during sample loading from HP chamber to quartz tube. Diffusion analysis is conducted by selecting only hydrogen peak emitted from rubber.

**Figure 4:**
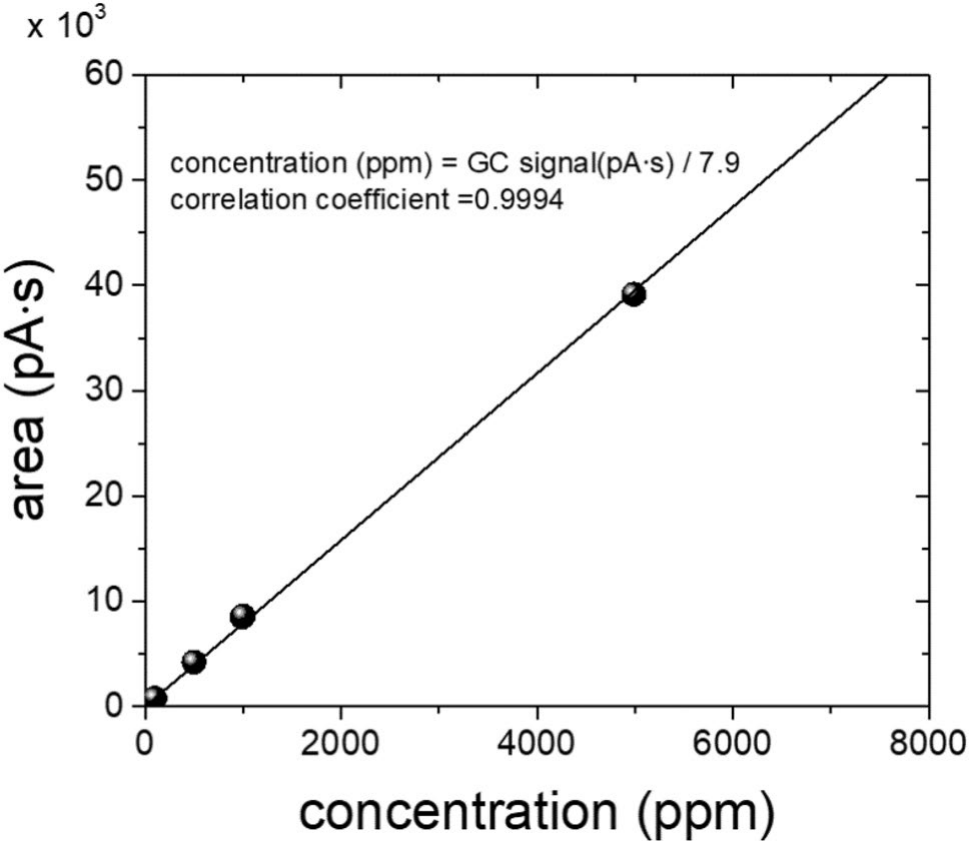
Calibration curve measured with standard hydrogen gases with concentrations of 100, 500, 1000, and 4989 ppm (balanced with helium gas).

To determine the molar concentrations that correspond to the areas of GC signals [pA·s] for the H_2_, we produced a calibration curve (Fig. 4) using standard H_2_ gas with known concentrations. The curve was linear with a slope of 7.9 pA·s/ppm and an intercept of 0. Thus, the area of the hydrogen gas GC signal in pA·s can be transformed to the molar concentration (mol ppm or just ppm) of hydrogen gas in the injected gas which is a mixture of hydrogen gas and balancing gas, here the helium gas using Eq. (2).

2 $$\mathrm{concentraion of hydrogen gas }\left(\mathrm{ppm}\right)=\frac{\mathrm{area of }{H}_{2}\mathrm{ GC signal }(\mathrm{pA}\cdot \mathrm{s})}{7.9}$$

### Hydrogen mass concentration from TDA-GC

Calculation of absolute mass of H_2_ charged in a sample from the GC measurement requires the information about flow rate of the carrier gas in GC system, the GC sampling volume *V*, the temperature *T*, and the pressure *p* of the sample in the sample loop. The released gas is injected into the GC system at a flow rate of 10 sccm at room temperature (298 K). Assuming an ideal gas ($pV=nRT$) under a constant *V* and *T*, the total number *n* of moles of the mixed gas is calculated by substituting the volume of the sample that is collected in the sample loop and sent to the GC column (*V* = 0.25 mL = 2.5 × 10^–7^ m^3^) and the gas constant [*R* = 8.20544 × 10^–5^ m^3^ atm/(mol K)] at 1 atm and 298 K as

3 $$n=\frac{1\mathrm{ atm}\cdot 2.5\times {10}^{-7}{m}^{3}}{(8.20544\times {10}^{-5}{m}^{3}\cdot \frac{\mathrm{atm}}{\mathrm{mol}\cdot \mathrm{K}}\cdot 298 \mathrm{K})}\cong 1.0224\times {10}^{-5}\mathrm{ mol}.$$

If the GC-measured concentration is *C* ppm, the number of moles of H_2_ gas in the mixed gas becomes as below,

4 $${n}_{{H}_{2}}\cong C\mathrm{ppm}\cdot \left({n}_{{H}_{2}}+{n}_{He}\right)\cong C\mathrm{ppm}\cdot n\cong 1.0224\times {10}^{-11}C \mathrm{mol},$$

where ${n}_{{H}_{2}}$ is the number of moles of H_2_ and ${n}_{He}$ is the number of moles of He. Equation (4) yields the total number of moles of charged H_2_. The mass of charged H_2_ can then be calculated with molar mass 2.018 g. Under experimental consideration of H_2_ concentration (maximum 1%) in He balanced gas, the effects on nitrogen and oxygen gas of 1% contained in carrier He gas could be neglected to be less than 0.1% in Eq. (4).

**Figure 5:**
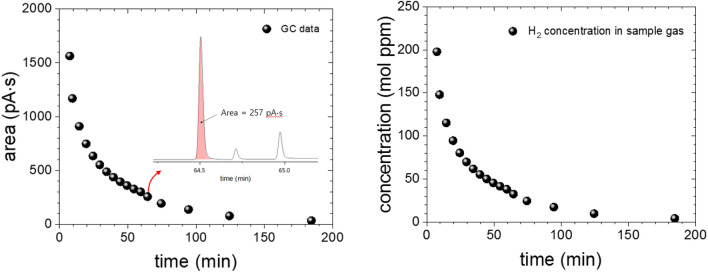
(Left) GC signal area versus the elapsed time after decompression. Inset is a set of GC spectra (peaks left to right: H_2_, O_2_, N_2_) measured for the injection at the time 63 min. The peaks for O_2_ and N_2_ are due to the air remaining inside quartz tube. The peaks corresponding about 2–3 ppm are eliminated through purge process with helium gas. (Right) H_2_ molar concentration converted from the GC raw data.

**Figure 6:**
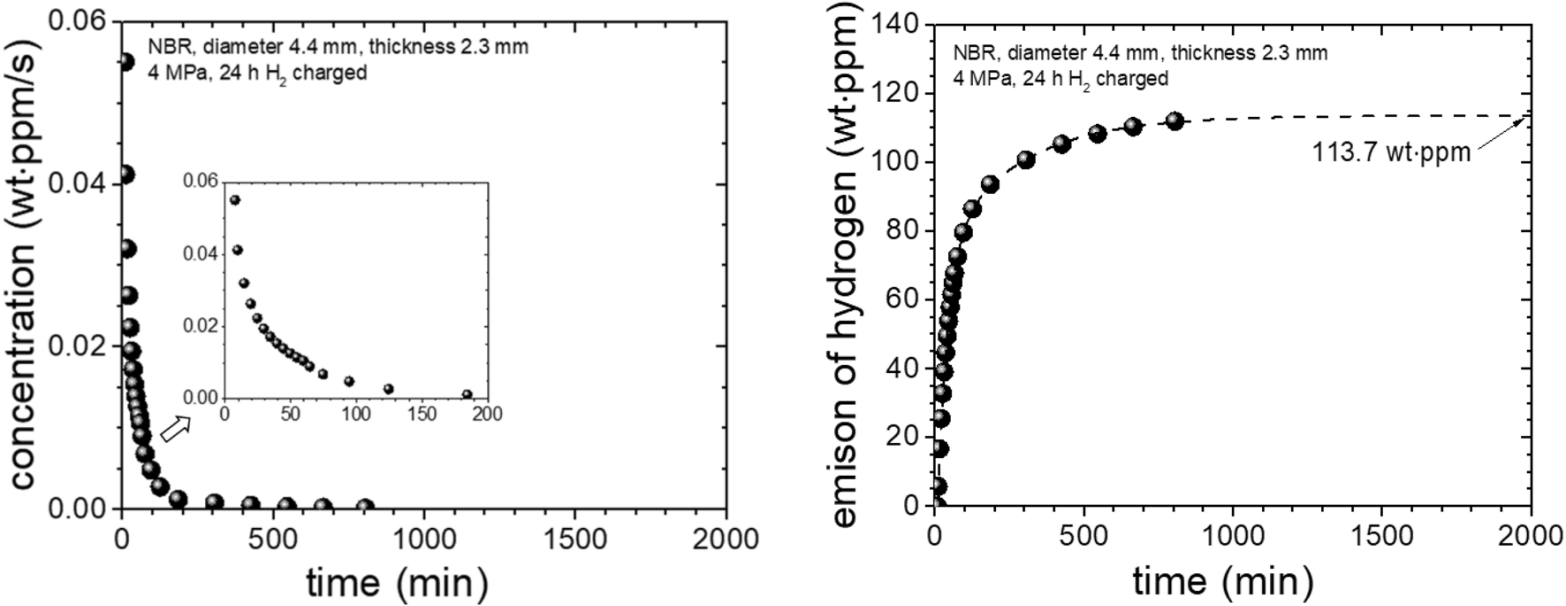
(Left) Time-dependent H_2_ concentration curve of an NBR sample expressed in mass concentration per second. (Right) Integrated curve of the time-dependent H_2_ concentration over time. The asymptotic value of 113.7 wt·ppm is obtained by extrapolating the integrated values using exponential functions.

**Figure 7:**
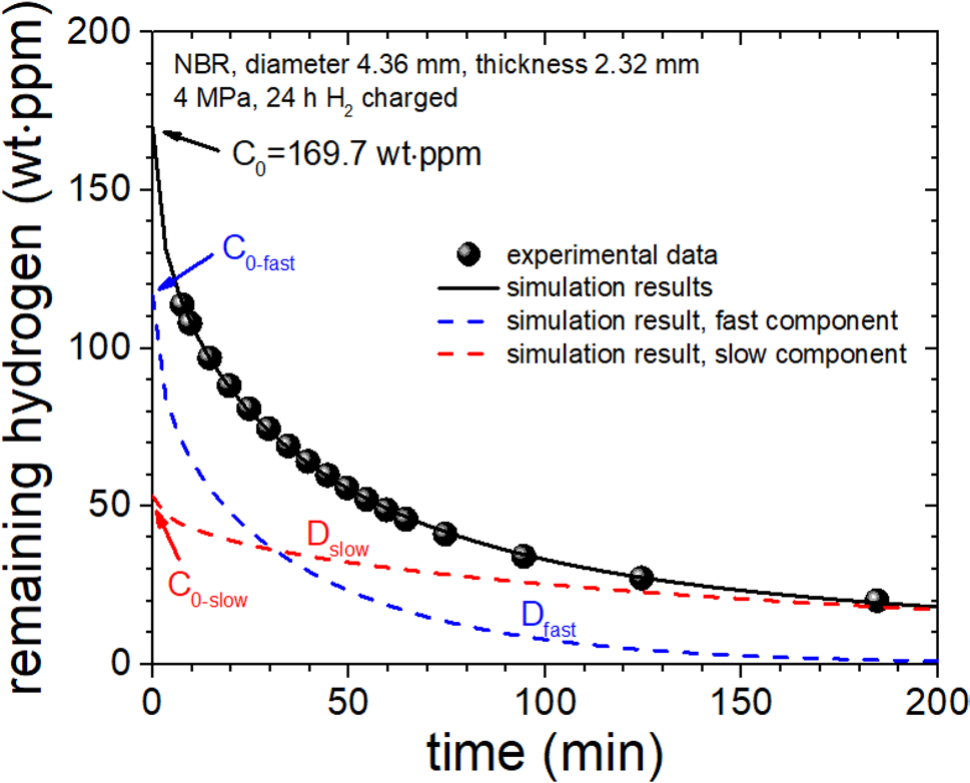
H_2_ residue data and simulation results over time after decompression with program. After parameter fitting using self-developed program, the graph was redrew with the experimental and simulation data using two components diffusivities, *D*_fast_ and *D*_slow_ and H_2_ residue *C*_0-fast_ and *C*_0-slow_.

From Figs. 5, 6 and 7 shows the process to obtain diffusion parameters of the H_2_ gas within an NBR sample with a diameter of 4.4 mm, a thickness of 2.3 mm, and a mass of 0.0494 g exposed to H_2_ gas at HP = 4 MPa for 24 h. From the GC measured raw data (Fig. 5, left), a molar concentration converted corresponding to each measurement was recorded as in right of Fig. 5 using Eq. (2).

Each molar concentration *C*_mol_ [mol ppm] in the figure is the H_2_ concentration within the gas which is injected into the GC sample loop with volume of 2.5 × 10^–7^ m^3^. Once this value is converted to the number of H_2_ moles by Eq. (4), it can also be converted to a mass by multiplying by the molar mass of H_2_. Since the time required to fill the 0.25 mL sample loop at a flow rate of 10 sccm is 1.5 s, we obtain a value in mass concentration per second for each GC signal by dividing the data in right of Fig. 5 by 1.5 s, and by the mass *m*_sample_ = 0.0494 g of the sample. The converted value indicates the mass concentration of H_2_ *C*_mass_ [wt·ppm] released per second for each GC measurement for one injection. The process above can be summarized as

5 $${C}_{\mathrm{mass}}\left(\frac{\mathrm{wt}\cdot \mathrm{ppm}}{\mathrm{s}}\right)={C}_{\mathrm{mol}}\left(\mathrm{mol}\cdot \mathrm{ppm}\right)\times \left\{1.0224\times {10}^{-5}\left(\mathrm{mol}\right)\right\}\times \left\{\frac{{m}_{{H}_{2}}(\frac{\mathrm{g}}{\mathrm{mol}})}{{m}_{\mathrm{sample}}(\mathrm{g})}\right\}\times \left\{\frac{1}{1.5 s}\right\},$$

and these values decrease exponentially over elapsed time (Fig. 6, left). Integrating with respect to time and extrapolating the results to infinite time yield the amount of H_2_ released from the moment at which GC measurement starts after decompression. The result was 113.7 wt·ppm (Fig. 6, right), which is obtained from the measurement at t = 9 min after decompression due to time lag. Thus, the amount of hydrogen emitted from t = 0 min to t = 9 min after decompression are missing. Thus, we have recovered the value of C_0_ as following steps.

To apply Eq. (1), the remaining amount of H_2_ should be obtained by subtracting the values of each point in right of Fig. 6 from 113.7 wt·ppm. By substituting the remaining amount of H_2_ at each time into Eq. (1) and using least squares regression to obtain *D* and *C*_*0*_ through the diffusion analysis program(Fig. 7), two H_2_-diffusion components were identified: a fast one and a slow one as shown in the figure. The fast diffusion had *D*_fast_ ≈ 1.17 × 10^–10^ m^2^/s, *C*_0-fast_ ≈ 116.6 wt∙ppm, and the slow diffusion had *D*_slow_ ≈ 1.7 × 10^–11^ m^2^/s, *C*_0-slow_ ≈ 53.1 wt·ppm. Overall, charging of H_2_ for 24 h at 4 MPa into a cylindrical NBR sample drove a total of *C*_0_ = 169.7 wt·ppm of H_2_ into the NBR, which value corresponds the contents of remaining hydrogen at 0 min by extrapolating the simulation line (black line) or corresponds the sum of two remaining hydrogens at 0 min by extrapolating two simulation results in both fast (blue dashed line) and slow (red dashed line) components, as shown in right of Fig. 7. By comparing the above diffusion coefficients with values previously reported^26^, and according to an explanation discussed in a later section, we can tentatatively interpret these results as follows. The component with a large (fast) diffusion coefficient is due to the H_2_ absorbed in the main macromolecular polymer that constitutes the rubber, and the small (slow) component is due to H_2_ absorbed in the carbon black (CB) filler.

The model that uses two diffusion rates had standard deviation = 1.1%, whereas that of the model that uses only one diffusion rate had standard deviation = 7%. This difference confirms that the use of two diffusion rates is superior to the use of one diffusion rate.

In similarity with NBR, by substituting the remaining amount of H_2_ at each time into Eq. (1) we determine *D* and *C*_*0*_ through the diffusion analysis program for EPDM and FKM cylindrical samples.

### Uncertainty analysis

The standard uncertainty factor of the TDA-GC method were evaluated according to the GUM^25^. There are two kinds of uncertainty which we should consider, i.e., type A, ${u}_{A}$ standard uncertainty caused by repeated measurements, and type B, ${u}_{B1}$…${u}_{B6}$ uncertainty, for diffusivity and solubility as follows.

i) Type A standard uncertainty ${u}_{A}$ by repeated measurements
ii) Type B uncertainty ${u}_{B1}$ due to the inaccuracy of the electronic balance
iii) Type B uncertainty ${u}_{B2}$ due to the linear drift of GC
iv) Type B uncertainty ${u}_{B3}$ due to the uneven diameter and thickness of the sample to be measured after decompression
v) Type B uncertainty ${u}_{B4}$ due to the standard deviation between test results and Eq. (1)
vi) Type B uncertainty ${u}_{B5}$ due to the inaccuracy of the analog manometer
vii) Type B uncertainty ${u}_{B6}$ due to the limited resolution of the analog manometer.

The standard uncertainty factors are uncorrelated and are independent, so the sensitivity coefficient is 1. Therefore, the combined standard uncertainty (${u}_{c}$) for *S* and *D* is expressed as a root sum of squares of the standard uncertainty factors:

6 $${u}_{c}=\sqrt{{u}_{A}^{2}+{u}_{B1}^{2}+{u}_{B2}^{2}+{u}_{B3}^{2}+{u}_{B4}^{2}+{u}_{B5}^{2}+{u}_{B6}^{2}}$$

The expanded uncertainty ($U$) can be expressed as the product of the coverage factor ($k$) and the combined standard uncertainty:

7 $$U=k\cdot {u}_{c}.$$

Uncertainty factor, combined standard uncertainty, and expanded uncertainty were summarized in Table 2 for *D* and *S* of H_2_ in the NBR sample.

**Table 2:** Uncertainty budget [%] for the diffusivity and solubility of NBR (diameter: 4.4 mm, thickness: 2.3 mm).

Permeation characteristics	Uncertainty factor							${u}_{c}$	$k$	*U*
	${u}_{A}$	${u}_{B1}$	${u}_{B2}$	${u}_{B3}$	${u}_{B4}$	${u}_{B5}$	${u}_{B6}$			
**Diffusivity**										
Fast	5.1	0.1	3.0	2.9	1.3	0.6	1.0	6.8	2.0	13.7
Slow	6.0	0.1	3.0	2.9	1.3	0.6	1.0	7.5	2.0	15.0
no filler	5.5	0.1	3.0	2.9	1.1	0.6	1.0	7.1	2.1	15.0
**Solubility**										
Fast	10.7	0.1	3.0	2.9	1.3	0.6	1.0	11.6	2.0	23.3
Slow	7.8	0.1	3.0	2.9	1.3	0.6	1.0	9.0	2.0	18.1
no filler	8.3	0.1	3.0	2.9	1.1	0.6	1.0	9.5	2.0	19.0

The uncertainties for *D*, *S* and *P* of H_2_ of an NBR sample with a diameter of ~ 10 mm and a thickness of 2.5 mm were also obtained by the same method, together with those of EPDM and FKM, which are shown as error bars in the following figure. The expanded uncertainty lies in the range from 13 to 27% for NBR, 11–23% for EPDM and 9–14% for FKM. The large expanded uncertainty is due to the Type A standard uncertainty. The larger uncertainty factors are of type A, that is, repeated measurements which may originate from the inhomogeneity among samples, type B uncertainties due to linear drift of GC and changes in the dimension of the sample. Uniform samples should be used to reduce the standard type A uncertainty originating from inhomogeneity among samples.

## Results and discussion

When manufacturing rubbers such as NBR, EPDM and FKM, large quantities of CB are added as reinforcing agents, additives, and fillers to improve the thermal, electrical, and physical properties. The CB used as a filler has different H_2_ absorption and permeation characteristics than the rubber matrix does, so the CB affects H_2_ gas behavior after decompression. These different behaviors can be deconvoluted by analyzing the amount of residual H_2_ over time with the help of developed program.

**Figure 8:**
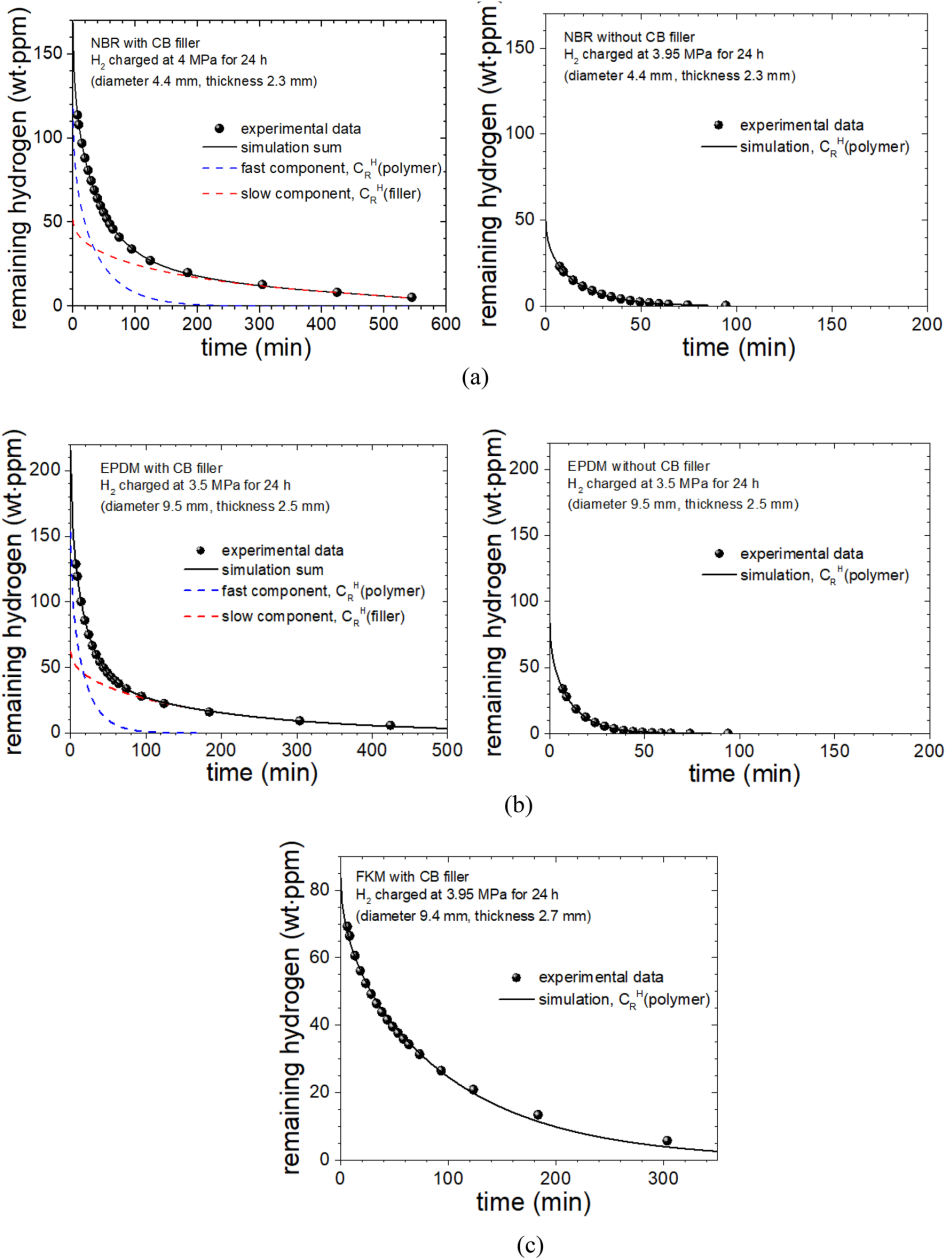
(**a**) Behaviors of remaining H_2_ contents versus time in NBR sample with CB filler (left) and without CB filler (right). (**b**) Behaviors of remaining H_2_ contents versus time in EPDM specimen with CB filler (left) and without CB filler (right). (**c**) Diffusion behaviors of residual H_2_ contents over time in FKM sample with CB filler. Black solid line is the fitted line using one diffusivity with a single Eq. (1).

The left and right sides of Fig. 8a show the amount of remaining hydrogen for NBR samples with and without CB fillers, respectively, where filled circles are the measurement result. On the left side of Fig. 8a, the black solid line is the simulation sum of the blue dotted line (fast diffusion) and red dotted line (slow diffusion), which are the two simulation results using the diffusion program shown in Fig. 7 by two Eq. (1). Description of the remaining H_2_ content of the NBR sample with CB filler required use of a fast diffusion coefficient and a slow diffusion coefficient (Fig. 8a, left]. Meanwhile the description for the NBR sample without CB filler could be fitted (Fig. 8a, right] using one diffusivity with a single term of Eq. (1) consistent with the experimental data, which corresponds to the fast diffusion coefficient in NBR with CB filler. Therefore, in NBR with CB, the component with a fast diffusion coefficient [C_R_^H^(polymer)] is due to the H_2_ absorbed in the main macromolecular polymer network that constitutes the rubber irrespective of CB filler, and the slow component [C_R_^H^(filer)] is due to H_2_ absorbed in the CB filler.

In similarity with NBR, Fig. 8b shows the time dependent content of remaining hydrogen in EPDM specimens. Filled circle is measurement result in EPDM with and without CB fillers. In the left part of Fig. 8b, black line is simulated sum of blue dotted line and red dotted line, which are two simulation contributions by two Eq. (1). The blue dotted line is explained as the fast diffusion of the specimen according to the H_2_ behavior in the polymer network, whereas red dotted line is the comparatively slow diffusion owing to the H_2_ trapped in the CB. As presented in left part of Fig. 8b, the permeation property of H_2_ in specimen was also explained as coming from two behaviors of H_2_ sorbed in rubber network and CB. In specimen without CB, the simulation [black line in right part of Fig. 8b with a diffusion behavior in one term of Eq. (1) are essentially in agreement with the experimental result.

However, the results simulated (black solid line) by the program with a diffusion behavior by a single term of Eq. (1), as shown in the FKM of Fig. 8c, were in agreement with the experimental result within standard deviation of 1%. The possibility for one diffusion behavior may be proposed by the following explanation. In the previous measurement of the FKM rubber filled with CB using precise electronic balances, the mass of FKM before and after hydrogen charging was compared with each other. The mass after hydrogen charging was taken at the attainment of equilibrium after hydrogen release from rubber at infinite time. It was found that the mass after hydrogen charging does fully not recovered to the mass before hydrogen charging, in other words, the mass after charging increased by ~ 40 wt∙ppm of mass before charging. This means that the part of penetrated hydrogen gas may remain in specimen and is replaced by interstitials or vacancies. However, the mass of the NBR and EPDM filled with CB before and after hydrogen charging are found to be same, implying most adsorbed hydrogen into rubber was desorbed from it.

Meanwhile, the contents of CB in FKM are less than those in NBR and EPDM, as shown in Table 1. The analogy is also found in SEM image for FKM. Therefore, one diffusion behavior due to no appreciable adsorption on the CB in FKM may appear by the smaller contents of fillers compared with NBR and EPDM. Because of this two reasons, a single hydrogen diffusion behavior in FKM may be only observed with a fast diffusion of polymer network instead of a slow diffusion adsorped in the filler.

**Figure 9:**
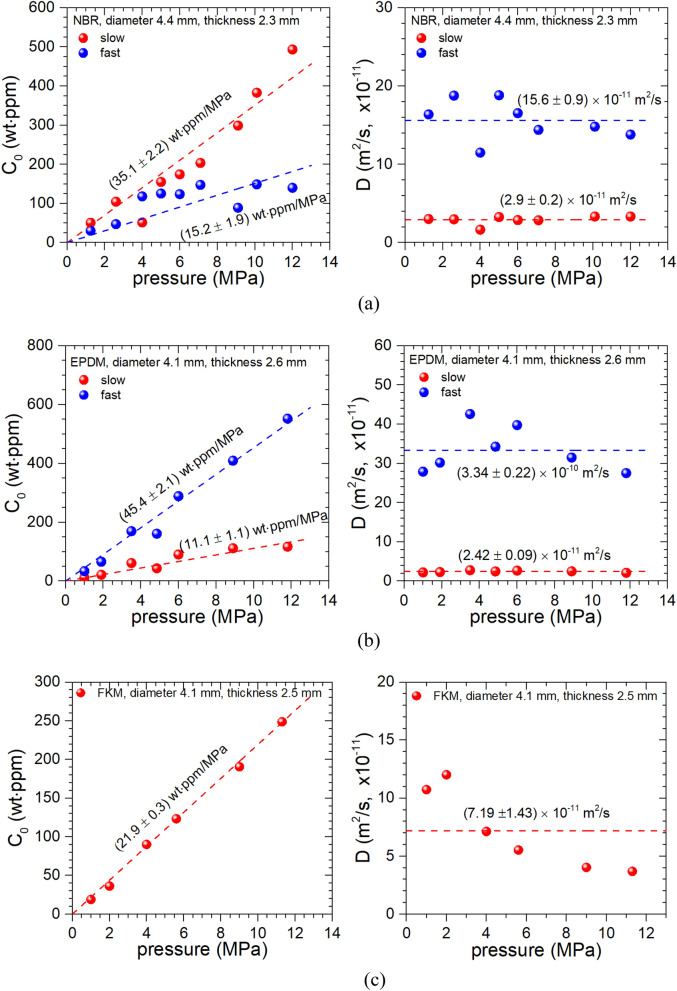
H_2_ content (left) and diffusivity (right) of (**a**) NBR, (**b**) EPDM and (**c**) FKM versus pressure.

To examine the permeation characteristics of the rubbers, we charged H_2_ for 24 h at pressures from 0.2 to 12 MPa to measure and analyze the release of H_2_ over time after decompression. Figure 9 show the *C*_0_ and *D* data with respect to the pressure in the cylindrical samples of NBR, EPDM and FKM with a radius of ~ 5 mm and a thickness of ~ 2 mm. Two different diffusion behaviors in NBR, EPDM and a single diffusion behavior in FKM for hydrogen content and diffusivity are shown for pressure up to 12 MPa, as mentioned above. The hydrogen content approximately follow Henry’s law. The solubility (*S*) of the hydrogen dissolved into rubber was calculated from the slope of left on Fig. 9 as following equation;

8 $$\mathrm{Solubility }\left(S\right)=\frac{\mathrm{slope }\left[\frac{wt\cdot ppm}{MPa}\right]{10}^{-6}\times d[\frac{g}{{m}^{3}}]}{{m}_{{H}_{2}}[\frac{g}{mol}]}$$

where *m*_H2_ is the molar mass of hydrogen *m*_H2_(g/mol) = 2.018 g/mol, and *d* is the density of rubbers used.

In the NBR and EPDM samples with CB filler on the right side of Fig. 9a, b, respectively, we observed two hydrogen diffusion behaviors, while FKM on the right side of Fig. 9c showed only one diffusion behavior. Since the observed diffusivity in the NBR and EPDM does not show pressure-dependent behavior, its representative value for various pressures is taken as the average value, as shown in right side of Fig. 9. Although FKM is observed the change of diffusivity with increasing the pressure, its representative value is also taken as the average value.

It is proposed that the pressure dependent behavior on diffusivity for FKM could be interpreted by the result of combination of Knudsen below 2 MPa and bulk diffusion above 2 MPa, which is observed and analyzed by fractal theory-based approach in the other researches^39,40^. In the case of fast component of NBR and EPDM in Fig. 9, the behaviors of similar pressure dependent diffusion observed was also observed. The bulk diffusion coefficient above 2 MPa is inversely proportional to pressure associated with mean free path between H_2_ molecules, whereas the Knudsen diffusion below 2 MPa normally occurs for the case with a large mean free path of diffusing gas molecules or its low gas density. Our data of cylindrical FKM with different diameter also shows the similar pressure dependence. However, we did not observe the pressure-dependent diffusivity in the spherical shaped FKM, which may be also associated with the shape of specimen used. The increase of the pressure may cause the decrease of the mean free path between molecules, resulting in the decrease of the diffusion coefficient.

**Figure 10:**
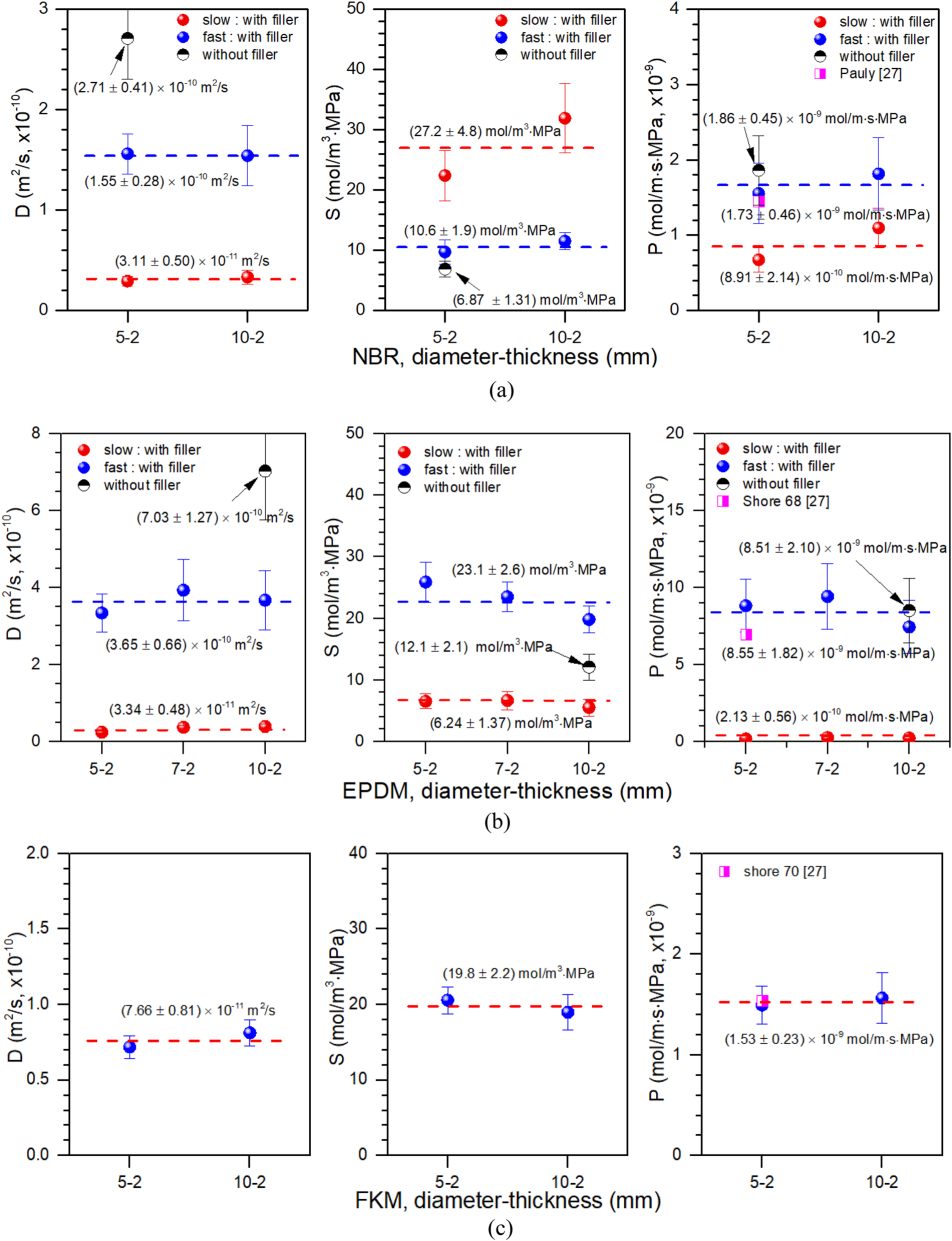
(**a**) Permeation characteristics according to the sample diameter in NBR. Left: diffusivity, middle: solubility, right: permeability. Error bars: expanded uncertainty estimated in a previous work. (**b**) Permeation characteristics according to the sample diameter in EPDM. Left: diffusivity, middle: solubility, right: permeability. Error bars: expanded uncertainty estimated in a previous work. (**c**) Permeation characteristics according to the sample diameter in EPDM. Left: diffusivity, middle: solubility, right: permeability. Error bars: expanded uncertainty estimated in a previous work.

The permeability (*P*) was obtained by multiplying the average diffusivity (*D*_*ave*_) for *D* at each charging pressure through a program simulation and solubility (*S*), i.e., *P* = *D*_*ave*_*S*. Figure 10 depicts the permeation parameters versus sample diameter for three rubbers. The permeation characteristics of the NBR sample (Fig. 10a) were obtained using samples with diameters of ~ 5 mm and 10 mm, but the same thickness ~ 2 mm. In the NBR sample with CB filler, two *D* values for fast and slow component were not significantly affected by sample diameter (Fig. 10a, left). Measured *D* of the NBR sample without CB filler (Fig. 10a, left) was faster than that of the fast component of NBR with CB filler. That is, filler-free samples of NBR had higher *D* than any of the samples that included filler; this difference implies that the H_2_ molecules adsorbed in the rubber matrix diffuse faster than those in the filler.

*S* was also not significantly affected by sample diameter (Fig. 10a, middle). However, *S* was higher in the slow component by CB than that in the rubber matrix. The *S* of NBR without filler is smaller than that two diffusivity of NBR with filler. As a result, the CB presence in NBR causes decrease in *D* and increase in *S*. The diffusion for NBR with CB filler in^26^ showed a single diffusion behavior with *S* = 32 mol/(m^3^ MPa), which coincides with S [27.2 ± 4.8 mol/(m^3^ MPa)] of the slow diffusion of our NBR with CB filler.

*P* was also unaffected by sample diameter (Fig. 10a, right). H_2_ with fast *D* had higher *P* than did H_2_ with slow *D*. Previous reported *P*^27^ had consistent value to both the *D* of NBR without filler and the fast-*D* component of NBR with filler in this study.

Figure 10b shows all the permeation parameters of the EPDM irrespective of the sample diameter. The left side of Fig. 10b shows the diffusivity of the fast and slow components. The diffusivity of the EPDM sample without filler was measured to be faster than that of the fast component with filler. In the middle of Fig. 10b, more hydrogen was dissolved in the fast component than in the slow component. The solubility for EPDM is consistent with that obtain by other group^26^. As shown in right on Fig. 10b, the permeability of hydrogen with fast diffusivity is greater than that of hydrogen with slow diffusivity, as expected. As a result of comparing the permeability of EPDM sample with a shore hardness of 68^19^, we can see that the values were consistent to those obtained for a large permeability of the fast component for the EPDM sample with filler and for the EPDM sample without a filler in our study.

As shown in the diffusivity results on the left of Fig. 10a, b, both NBR and EPDM samples without fillers are had *D* that was approximately twice as large as those of the fast components of NBR and EPDM with fillers. This result indicates that the fast component shows the permeation characteristics of H_2_ adsorbed onto the parent component of the rubber, and the slow component shows the permeation characteristics of H_2_ adsorbed to the filler. These findings are consistent with the analysis in a previous study^26^.

As shown in Fig. 10c, the permeation characteristics for single component in the FKM were not dependent on the sample diameter. The right side of Fig. 10c shows the reference value of an FKM sample with a shore hardness of 70 measured in a prior study^27^, and we can see that the value is similar to the values obtained in this study.

In summary, the permeability (P) was obtained with the magnitude in the order P_EPDM_ > P_NBR_ > P_FKM_. The major properties for the sealing material are volume swelling after decompression, penetration amount of H_2_ into rubber under high pressure, glass transition temperature (T_g_) and leakage after the cyclic testing. The correlations between these associated parameters is under study. Especially, EPDM rubber is an appropriate candidate for seals materials in the low temperature for because of low T_g_. The domestic company is applying to the EPDM materials and developing to for use in hydrogen vehicle.

In next work, the correlation between gas permeation and diffusion coefficient should be studied for one polymer with different filler contents, that is, the effect of filler concentration on hydrogen permeation is conducted as a systematic manner.

## Conclusions

We have investigated the permeation characteristics of H_2_ gas by quantifying and analyzing the amount of H_2_ gas released after decompression, by using TDA-GC and a diffusion-analysis program. After measuring the change in the absolute mass of H_2_ gas released from the rubber, we established a precise technique to determine the amount of charged H_2_ and its diffusivity and obtained the solubility by using Henry’s law and permeability by calculating *P* = *S*·*D*. This is the first report to apply this technique to a cylindrical rubber samples to evaluate the full permeation characteristics of H_2_ gas according to changes in both pressure and sample size.

Investigations on three rubbers represented that the permeation properties (*S*, *D*, *P*) of H_2_ are not appreciably dependent on specimen diameter investigated on this study. Hydrogen in the NBR and EPDM has two behaviors of diffusion, that is, fast diffusion owing to the hydrogen adsorbed in the polymer chain and slow diffusion owing to the H_2_ trapped in the CB filler. Origin for two diffusion behaviors for NBR and EPDM was discovered by comparing diffusion coefficient versus time for specimen including CB filler with those without CB filler. On the other hand, FKM has a single hydrogen diffusion behavior in the polymer. The charged H_2_ content under a pressure up to 12 MPa could be interpreted by Henry’s law. This indicates that the amount is substantially proportional to the charging pressure. The evaluating results of the permeability of three rubber samples were in agreement with the results of previous researches within the expanded uncertainty magnitude, thereby validate this method established in present investigation.

TDA-GC is a sophisticated technique for observing H_2_ behaviors and can obtain the respective diffusivities by separating two or more behaviors from mixed H_2_ groups. Thus, TDA-GC was successfully used for the analysis of multicomponent gas permeation. This method can detect the amount of H_2_ gas charged in an even small sample by converting the GC-measured electrical signals by the PDD method and measuring the absolute mass of H_2_ gas by using standard H_2_ gases traceable to national standards. From the quantitative analysis of parameters (*C*_0_, _polymer_, *D*_polymer_ and *C*_0_,_filler_, *D*_filler_) obtained from two different diffusion behaviors in NBR, we could estimate the magnitude of the effects of both polymer and filler on the permeation properties. However, further research is required to reduce the type A uncertainty by using an even-uniformity sample and performing the inter-comparison with abroad group.
